# Implementation of Enhanced Recovery After Surgery: a strategy to transform surgical care across a health system

**DOI:** 10.1186/s13012-017-0597-5

**Published:** 2017-05-19

**Authors:** Leah M. Gramlich, Caroline E. Sheppard, Tracy Wasylak, Loreen E. Gilmour, Olle Ljungqvist, Carlota Basualdo-Hammond, Gregg Nelson

**Affiliations:** 1 Department of Medicine, University of Alberta, Edmonton, Canada; 2 Department of Surgery, University of Alberta, Edmonton, Canada; 3 Alberta Health Services, Edmonton, Alberta Canada; 4 Department of Surgery, Faculty of Medicine and Health, School of Health and Medical Sciences, Örebro University, Örebro, Sweden; 5 Tom Baker Cancer Centre, Department of Oncology, University of Calgary, 1331 29 Street NW, Calgary, Alberta Canada; 6 Gastroenterology, Royal Alexandra Hospital, 214 CSC, 10240 Kingsway Avenue NW, Edmonton, AB T5H 3V9 Canada

**Keywords:** Enhanced Recovery After Surgery, Implementation, Theoretical Domains Framework, QUERI

## Abstract

**Background:**

Enhanced Recovery After Surgery (ERAS) programs have been shown to have a positive impact on outcome. The ERAS care system includes an evidence-based guideline, an implementation program, and an interactive audit system to support practice change. The purpose of this study is to describe the use of the Theoretic Domains Framework (TDF) in changing surgical care and application of the Quality Enhancement Research Initiative (QUERI) model to analyze end-to-end implementation of ERAS in colorectal surgery across multiple sites within a single health system. The ultimate intent of this work is to allow for the development of a model for spread, scale, and sustainability of ERAS in Alberta Health Services (AHS).

**Methods:**

ERAS for colorectal surgery was implemented at two sites and then spread to four additional sites. The ERAS Interactive Audit System (EIAS) was used to assess compliance with the guidelines, length of stay, readmissions, and complications. Data sources informing knowledge translation included surveys, focus groups, interviews, and other qualitative data sources such as minutes and status updates. The QUERI model and TDF were used to thematically analyze 189 documents with 2188 quotes meeting the inclusion criteria. Data sources were analyzed for barriers or enablers, organized into a framework that included individual to organization impact, and areas of focus for guideline implementation.

**Results:**

Compliance with the evidence-based guidelines for ERAS in colorectal surgery at baseline was 40%. Post implementation compliance, consistent with adoption of best practice, improved to 65%. Barriers and enablers were categorized as clinical practice (22%), individual provider (26%), organization (19%), external environment (7%), and patients (25%). In the Alberta context, 26% of barriers and enablers to ERAS implementation occurred at the site and unit levels, with a provider focus 26% of the time, a patient focus 26% of the time, and a system focus 22% of the time.

**Conclusions:**

Using the ERAS care system and applying the QUERI model and TDF allow for identification of strategies that can support diffusion and sustainment of innovation of Enhanced Recovery After Surgery across multiple sites within a health care system.

## Background

The Enhanced Recovery After Surgery (ERAS)® Society evidence-based guidelines for colorectal surgery encompass care before, during, and after surgery, following the patient on their surgical journey, to support early recovery. The guidelines bundle 22 interventions, which, when implemented together as part of a multimodal, multidisciplinary perioperative care pathway, result in accelerated recovery, decreased perioperative stress, pain and gut dysfunction, and reduced severity of complications for patients undergoing major surgery [1–3]. The accelerated recovery and reduced complications are associated with a reduction in length of stay (LOS) in hospital, a reduction in total complication rates, and no increased burden on primary care or emergency department usage [1, 2, 4]. Patient-reported outcomes, including pain and fatigue, are improved with ERAS® evidence-based guideline implementation, and the reduction in length of stay in hospital does not appear to have an adverse effect on quality of life and patient satisfaction [5, 6].

Despite the robust evidence in support of ERAS, the ERAS® evidence-based guidelines have not been widely adopted. These ERAS® evidence-based guidelines challenge deeply rooted and dogmatic perioperative practices related to fasting, pain management, and mobilization. Many of the clinical practice changes called for under the ERAS colorectal protocol are radical in the sense that they involve stopping certain long-standing practices, such as no bowel prep before colorectal surgery and fasting after midnight, in many cases, replacing those practices with a seemingly contrary practice such as carbohydrate loading before surgery.

The extent to which all of the 22 recommendations in the ERAS® evidence-based guidelines are implemented impacts success in driving improvement in patient, system, and economic outcomes [7, 8]. Research is lacking on how to most efficiently put knowledge into practice integrated knowledge translation (iKT) into action, particularly for such a complex and multifaceted intervention as the ERAS® colorectal evidence-based guideline.

### Context: ERAS implementation in Alberta Health Services

Alberta Health Services (AHS) is a provincial health system responsible for the provision of surgical care to over three million Albertans at 57 surgical sites with an annual budget of over one billion dollars. In 2013–2014, AHS implemented the ERAS® colorectal protocol in six unique sites performing over 75% of all colorectal surgeries in the province as a proof-of-concept project [9]. The QUERI (Quality Enhancement Research Initiative) approach was used [10] to provide the most appropriate and efficient way to implement Enhanced Recovery across AHS. This consisted of the following steps: (1) identifying high-risk/high-volume diseases or problems (e.g., colorectal surgical care), (2) identifying best practices (e.g., ERAS® evidence-based guideline), (3) defining existing practice patterns and outcomes (e.g., “compliance” with the guideline), (4) identifying and implementing interventions to promote best practices (e.g., change management strategies centered on audit of practice), (5) documenting that best practices improve outcomes (e.g., change in compliance with the guideline, LOS, readmissions complications), and (6) documenting outcomes that are associated with improved health. In adopting the QUERI approach and in applying the ERAS® care system, we are able to detail end-to-end implementation of this complex intervention within and across a health system.

The ERAS® care system applied within each site included (1) the ERAS® evidence-based colorectal guideline [11], (2) the ERAS® Implementation Program (EIP) for change management, and (3) the ERAS® Interactive Audit System (EIAS). The EIAS is an Internet-based data entry and analysis system, which tracks and measures compliance with the evidence-based guideline by the site-based ERAS team. The EIP includes an implementation program for change management consisting of detailed coaching and supervision of an implementation team in “Train the Trainer” (TTT) sessions, including a surgeon as the local leader in practice, an anesthesiologist, and a nurse leader acting as the coordinator, at a given site in a particular surgical area. In addition to reinforcing practice change and tailored interventions using the PDSA (Plan-Do-Study-Act) cycle, local modifications to the EIP in AHS include the use of the learning collaboratives [12], which allow teams from across multiple sites to engage and share their learning. The EIAS is used by the site-based ERAS implementation teams to monitor compliance with the ERAS® evidence-based guidelines, to facilitate implementation by providing real-time feedback to teams based on all patients undergoing ERAS® care, and to support tailored interventions to accelerate uptake of practice change [11].

Planning for expanded implementation of the ERAS® Society evidence-based guidelines to other surgical areas in Alberta Health Services illuminated the need for a more informed approach. This approach was undertaken to improve our ability to support and predict the success of ERAS® implementation at a site and to detail strategies for successful implementation allowing spread, scale, and sustainability of Enhanced Recovery strategies more broadly across the health system.

Many theories and frameworks of behavior change exist, and often these theories have conceptually overlapping constructs [13–15]. Only a few of these theories have been tested in robust research in health care settings. Within the QUERI model, we adopted the Theoretical Domains Framework (TDF) [16], as it is a well-operationalized multilevel implementation determinant framework, to help conceptualize the implementation at multiple levels and generate practical and applicable findings related to strategies and interventions which impact outcomes [17]. We applied it at the individual and organizational levels in an exploratory fashion to identify barriers and enablers to this complex intervention. This will allow future research to map the results of TDF-based problem analysis onto intervention components and provide a solid rationale for spread and scale of our implementation interventions. The TDF seeks to identify who needs to do what differently to adopt best practice, what barriers and enablers need to be addressed, which intervention can be employed to overcome these barriers and enhance the enablers to uptake best practice, and to measure and understand behavior change. Psychological theories can be used within this framework to understand barriers to changing practice. The main strength of this four-step method is that it can be used as a guide for implementation intervention developers, because it is a systematic method of moving from target behaviors, to theoretical domains, to behavior change techniques, and finally to a full implementation intervention. The authors propose a streamlined approach moving directly from identified theoretical domains relevant to the implementation problem to behavior change techniques. Importantly, in this model, the delivery mode in the clinical setting for behavior change is guided by local context and what is acceptable and feasible in the target group. This meshes well with the ERAS® implementation program, which uses audit of compliance with an evidence-based guideline to drive practice change at the provider level [11] and which builds upon an implementation program for change management. There is flexibility in this approach to designing implementation interventions and combining research evidence, matrix mapping, and feasibility information that allows responsiveness to the local context.

The goal of this research is to develop a model for spread, scale, and sustainment of ERAS across a health system through the application of the QUERI model, the TDF, and development of a knowledge translation framework. The research question that is asked is: What are the barriers and enablers to ERAS implementation within a health care system?

## Methods

### ERAS implementation

The implementation of ERAS was staggered and began with two early adopter sites in mid-2013 where we garnered feedback, learnings, and insight prior to implementation at four additional sites in 2014. The active phase of implementation took 9–12 months. The ERAS® Society trained and supported the first two sites for the initial implementation phase with a series of implementation meetings which were undertaken initially to introduce the concept of ERAS and the tools, to review baseline data, and finally to review the data on the first 50 patients post implementation and review the interpretation and use of the data relative to practice change. These meetings brought together ERAS teams from across the province and also provided an opportunity for networking. With subsequent implementation in AHS, a locally tailored approach to the ERAS implementation was used. As we undertook implementation at each site, we applied the TDF and used mixed methods to systematically aggregate information to inform further implementation as it related to the following: who needs to do what differently, what were the barriers and enablers to practice change, what strategies were used to address barriers and enablers, and to measure behavior change and impact on outcome.

As part of the implementation program for change management, surgeons and anesthetists working within an EIP and ERAS team were identified within their respective sites based on their willingness and ability to champion ERAS. For each site-based ERAS team, nursing coordinator support was provided through the implementation plan. The site-based implementation teams worked within their own areas to create capacity, linking to unit and site management and educators. In addition, at the unit, site, zone, and provincial levels, we undertook regular communication and provided opportunities for feedback. Weekly meetings were held with site coordinators and project and provincial supports to discuss implementation, and problem solve barriers to implementation. At the provincial level, an ERAS Alberta Secretariat was appointed to provide leadership and direction and to link with provincial, national, and international strategies. This included physician leadership as well as leadership from Strategic Clinical Networks (SCNs) Provincial Nutrition and Food Services and Provincial Pharmacy Services of AHS. SCNs are the engines for change in the AHS health system, composed of researchers, physicians, patients, and managers working together to find new and innovative ways of delivering health care for Albertans [18]. Content from this group was aggregated on an ongoing basis.

We used the ERAS® evidence-based guideline for colorectal surgery [19] as the standard for best practice and compliance with the guideline as an indication of variance from “best practice” across all sites (e.g., who needs to do what differently). Prior to implementation, data on a baseline cohort of 50 patients at each site was used to define compliance with the ERAS evidence-based guideline preimplementation. The use of the EIAS and implementation of ERAS® in colorectal surgery in AHS has been previously described [9].

### Materials: data collection informing ERAS knowledge translation framework for system transformation

We collected data from a variety of stakeholders and knowledge users at the patient, provider, and system levels from 1 survey, meeting notes, 3 learning collaboratives, 2 focus groups, 4 interviews, status reports, and memos, which included input from 15 patients, 56 nurses, 13 clinical nurse educators, 1 unit clerk, 2 patient safety officers, 16 surgeons, 12 anesthetists, 6 dietitians, 31 unit managers, 1 occupational therapist, 1 physiotherapist, 1 enterostomal therapist, 33 AHS or site executives/managers, 6 site coordinators, 3 internal medicine doctors, 5 knowledge consultants, and 2 pharmacists. In total, 99 individuals participated in surveys that were distributed to their local units. We undertook 6 interviews and 1 focus group (8 participants) with patients who had undergone colorectal surgery within the ERAS implementation to address patient perspectives. These interviews were approved by the Health Research Ethics Board at the University of Alberta (Pro00046864). Refer to Table 1 for level-specific methods.

**Table 1:** Data collection methods and number of documents used per method to address patient-, provider-, and system-level change

Level	Data collection methods					
	Interviews	Focus groups	Surveys	Learning collaboratives	Status reports	Status reports and memos
Patient	6	1	–	3	2	25
Provider	2	2	1	3	28	31
System	2	2	–	3	32	52

### Data analysis

Qualitative data was aggregated into NVivo 11 (QSR International) from a variety of electronic sources available on an AHS SharePoint, a secure electronic data storage and sharing platform hosted on the AHS intranet. Inclusion criteria was staff surveys, meeting notes, learning collaborative notes, TTT session minutes, project evaluation reports, focus groups, interviews, and memos. These documents were analyzed for factors that inhibited, slowed, or were obstacles (barriers) and factors that facilitated, helped, or overcame obstacles (enablers) to care. Data from the six sites implementing the ERAS colorectal protocol was sent by the site coordinator to the researchers quarterly and was pulled from SCN and ERAS Alberta documents (e.g., ERAS Secretariat meetings). Exclusion criteria for documents included figures, data figures, PowerPoint presentations without words, and order sets. Sites sent their data to the ERAS research team 2 weeks after the end of every quarter. Data was locked by quarter once the documents were received. Data coding and analysis was undertaken by ERAS researchers with expertise in qualitative methodology. Word searches were undertaken to identify quotes as either a barrier (using similar word definition, e.g., problem, obstacle, issue) or an enabler (e.g., aid, help, easy) allowing analysis relating to various models and frameworks (TDF, QUERI, Rubenstein [17, 20]). Important words were explored by illustrating word frequencies as word clouds. First, each barrier and enabler was independently categorized into one of the 22 elements based on ERAS definitions [11]. One researcher (CS) went through the documents and coded for barriers and enablers, by element. A textual analysis [21] using open coding was performed. Categories for logbook, communication, videos, team/leadership, and data were added from feedback that interviewees indicated were important topics. Each quote was also coded into Rubenstein’s QUERI framework [20] for health care implementation. Because the health system in Alberta is different than that in the USA, three key categories were missing that were thought to be relevant to the analysis: system as Alberta has a single health care system, zone as Alberta is divided into five health zones, and unit as hospitals have different units. These categories were added to the analysis to enhance Rubenstein’s QUERI framework for Canadian research.

The data was analyzed using an inductive and an a priori deductive approach. We open-coded using Turner’s [22] category card method in order to reflect the data as close to its original state (i.e., verbatim) as possible. The deductive approach was developed before we analyzed the data (see Appendix 1). Using our in-depth knowledge about ERAS, we developed a list of categories based on the 22 ERAS elements and unit (e.g., system, provider). We tested these categories on quotes/segments from the textual data, revising them where necessary. Once the data was open-coded, we mapped the quotes onto the TDF and QUERI to assess gaps and opportunities. Quotes were stratified into the QUERI sections by the overarching theme of the quote.

Example 1: “In the meantime pharmacy is trying to set up a caution to look at the time of last dose. RAH has no issue because they have VAX. Very beneficial for ERAS.” A hospital electronic program (VAX) preventing administration of an extra thromboprophylaxis dose to patients is an enabler that fits into Organization of the QUERI framework.

Example 2: “Surgeons not buying into the program gets in the way—telling people things in the office vs. in hospital it ends up being conflicting advice. Now nurses ask “what has your doctor told you” as the starting point. Consistency is a challenge because the nurses and docs aren’t on the same page—it causes mistrust in the patient. Patient needs to know they are at the heart of it and that the team is working together around them to make their outcome a good one.” Provider buy-in and team dynamics is a barrier that fits into Provider of the QUERI framework.

We presented and discussed results of the preliminary analysis of barriers and enablers with the ERAS Alberta team to seek feedback and to resolve discrepancies. Barriers and enablers were coded into multiple categories to allow for the development of a model for spread, scale, and sustainability of ERAS in AHS. To understand the prominent themes, quotes were themed according to a predefined strategy (see Appendix 1), and separate word clouds were created based on word frequency.

Data was analyzed after all six participating sites had been in implementation for at least 12 months. NVivo 11 (QSR International) was used to house and analyze the knowledge translation (KT) data.

### Assessment of ERAS implementation: iterating towards guideline compliance

Based on locally identified barriers and enablers to best practice, site-based teams identified strategies to address these barriers and enablers to ERAS implementation. This was supported through the initial TTT sessions where teams were taught how to use the EIAS to identify areas for focus based on compliance, complications, and outcomes. The teams worked within their own sites to prioritize and to implement practice changes using the Plan-Do-Study-Act cycle. Site teams reviewed their EIAS data to identify areas of low compliance or non-compliance (e.g., early postoperative nutrition supplementation), formulated theories of causation (e.g., postoperative nausea, measured by EIAS), defined what success could look like (e.g., around-the-clock, appropriate antiemetic therapy), and then put the Plan into action (e.g., work with ward staff including pharmacists, nurses, and dietitians to create a local plan for implementation of around-the-clock antiemetic therapy). Following implementation (the Do phase), the ERAS site team would reassess compliance with early postoperative feeding and complications of postoperative nausea through EIAS to see if the Plan (process and plan for around-the-clock antiemetic therapy) had an impact as measured by increased compliance with early postoperative nutrition supplementation and reduction in postoperative nausea. The Plan would be Acted upon and the intervention would be formalized into action plans based on the EIAS reports. The site coordinators met regularly with direction and support from the ERAS Alberta team to discuss strategies across sites and to share experiences. This information was captured with meeting minutes. In addition, three scheduled learning collaboratives were undertaken. During these all-day sessions, all members of the site-based ERAS teams and the ERAS Alberta team reviewed current status and targeted performance goals related to specific aspects of ERAS implementation at their unique sites using performance benchmarks. These learning collaboratives also provided an opportunity for teams to network and to learn from one another. Detailed notes taken during these sessions were included in the analysis.

To identify what practice changes occurred, we assessed change in compliance with the ERAS® colorectal evidence-based guidelines (based on the EIAS), comparing baseline compliance prior to implementation to compliance with the guidelines post implementation. This data was refreshed quarterly and updated across all sites. Overall compliance was assessed as well as compliance in the preoperative, intraoperative, and postoperative time periods. To assess the impact of practice change on outcome, we used data from EIAS. Outcome evaluation included assessment of LOS, complications, readmissions, and cost. A two-sample *t* test was used to compare pre-ERAS and post-ERAS implementation for compliance and LOS.

Ethics approval and informed consent were obtained to undertake surveys, interviews, and focus groups with patients and providers from the Human Ethics Research Boards at the University of Alberta and University of Calgary.

## Results

### Context—implementation, impact on outcome

The context of this implementation work is provided through the published work on the AHS ERAS implementation to date. The ERAS® care program was applied in a total of 2587 consecutive patients (352 pre- and 2235 post-ERAS implementation) undergoing colorectal surgery between February 2013 and October 31, 2015, at six Alberta hospitals. The intervention had a positive impact on patient and health system outcomes and was effectively applied across multiple institutions. In brief, the median overall guideline compliance was 39% in pre-ERAS and 60% in post-ERAS patients. The median LOS was 6 days for pre-ERAS compared to 4.5 days in post-ERAS patients with the longest implementation. Complications and readmissions were reduced. The net cost savings attributable to guideline implementation ranged between US$2806 and US$5898 per patient [7, 9].

**Fig. 1:**
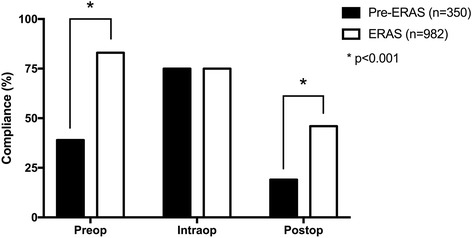
Compliance change before and after ERAS guideline implementation

The analysis of this data is an extension of the original data set. Initial overall compliance with the ERAS® evidence-based guideline at baseline (preimplementation) was 39% with the lowest compliance with postoperative (19%) guideline elements (Fig. 1) (*p* < 0.0001). After implementation, overall compliance significantly increased to 60%, with the greatest increase in compliance seen in the preoperative period (39 to 83%, *p* < 0.0001). The compliance at baseline and post implementation were similar across all six sites and were reflective of baseline practice, variance from “best practice,” and areas of focus for practice change within and across programs.

### Knowledge translation data analysis

**Fig. 2:**
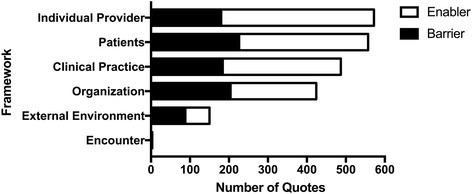
Number of total quotes based on QUERI [20]. Quotes are separated into barriers and enablers

**Fig. 3:**
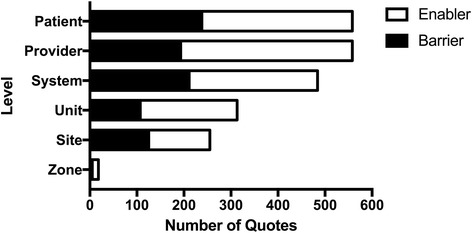
Total number of quotes by level. Quotes are separated into barriers and enablers

**Fig. 4:**
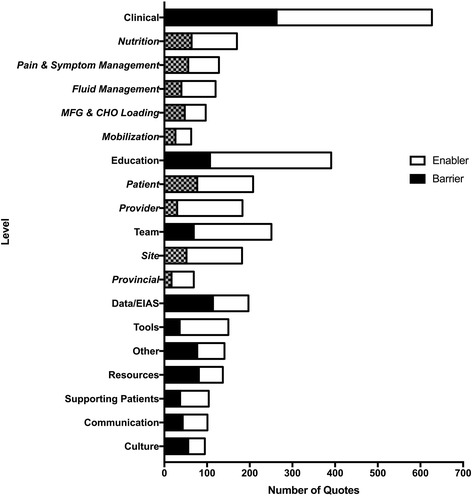
Total number of quotes stratified by “Spread and Scale” themes, discussed themes, noticeable themes, and other. Quotes are separated into barriers and enablers. *MFG & CHO Loading* modern fasting guideline and carbohydrate loading. Patterned: sub-theme of above theme

**Fig. 5:**
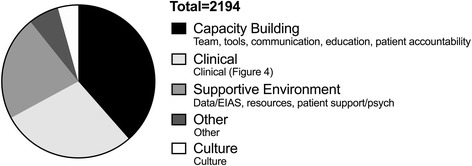
Total number of quotes related to sustainability [37]. Themes for “Spread and Scale” were stratified into “Sustainability” categories noted in inclusion. Quotes are separated into barriers and enablers

Qualitative data was aggregated as described above. A total of 925 documents were initially evaluated, and a total of 198 documents met inclusion criteria. A total of 2188 quotes were included from the documents meeting inclusion criteria. Documents met inclusion criteria if they contained text (i.e., no graphs, empty order sets, photos, or videos were included). The aggregate results are presented in Figs. 2, 3, 4, and 5. Table 2 illustrates ERAS care elements, which are driven by the evidence-based guideline Appendix 2. Qualitative analysis suggested that the areas in which there was more difficulty in implementation were often reflected by having a greater number of quotes. Factors generally thought to influence provider decisions and practices include characteristics of the external environment surrounding a health care organization, the health care organization itself, the clinical practice, the individual health care provider, the patient, and the encounter between clinician and patient [20]. Data analysis within these themes identified clinical practice (22%), patients (25%), individual provider (26%), organization (19%), and external environment (7%) in the quotes cited (Fig. 2). In the Alberta context, 26% of barriers and enablers to ERAS implementation occurred at the site and unit levels, with a provider focus 26% of the time, a patient focus 26% of the time, and a system focus 22% of the time (Fig. 3).

**Table 2:** ERAS 22 elements

ERAS 22 elements	
Preoperative	
1. PAC patient education	
1. PAC shared decision-making	
1. PAC nutrition	
1. PAC medical optimization	
2. Fluid and carb loading	
3. No prolonged fasting	
4. No/selective bowel prep	
5. Antibiotic prophylaxis	
Intraoperative	
6. Thromboprophylaxis	
7. No premedication	
8. Nausea and vomiting prophylaxis	
9. Short-acting anaesthetic agents	
10. No drains	
11. Avoidance of salt and water overload	
12. Maintenance of normothermia	
Postoperative	
13. Mid-thoracic epidural anesthesia/analgesic	
14. No nasogastric tubes	
15. Prevention of nausea and vomiting	
16. Avoidance of salt and water load	
17. Early removal of catheter	
18. Early oral nutrition	
19. Non-opioid oral analgesia/NSAIDs	
20. Early mobilization	
21. Stimulation of gut motility	
22. Audit of compliance and outcomes	
Not applicable	

In order to allow better insight into implementation issues related to sustainability, we applied QUERI considerations articulated by Stetler et al. [10]. The pooled data identifies capacity building, related to teams, tools, communication, and education, in 39% of the barriers and enablers studied, clinical elements in 24%, and supportive environment, defined as data-related issues, supporting patients, and human resource availability, largely related to nurse coordinators, in 22% of quotes (Fig. 4). Key themes were identified to allow for focus in consideration of diffusion and spread and scale of ERAS and included specific clinical components, education, teams, resources, tools, audit and data, and communication. Supporting patients was also identified as an important theme by both patients and health care providers (Fig. 5).

Clinical considerations of focus included nutrition, mobilization, diabetes, pain and symptom control, fluid management, modern fasting guidelines, and carbohydrate loading. This data was further sub-themed. Barriers to nutrition care related to processes at the sites, the nature of the oral supplements, and information and communication for patients related to eating and drinking after surgery. Strategies to enhance nutrition care built upon process improvement for ensuring delivery of appropriate snack and supplements and encouragement and education for patients and health care providers. Mobilizing patients was challenging for nursing staff who would have valued involvement of physiotherapy and from patients’ families. This was an area that was not well tracked with medical records. Aggregate involvement of all nursing staff, including nursing aides, and physiotherapy, and applying these standards to all patients on the unit, not just ERAS patients, were identified as opportunities. Fluid optimization in ERAS focuses on euvolemia with negative impact identified related to both dehydration (i.e., from bowel prep and prolonged preoperative fasting) and volume overload. Intraoperatively, anesthesiologists did not have equipment ready or consistent access to track volume status. In the recovery room and on the wards during recovery, volume was tracked by daily weights which were not recorded consistently. Surgical residents were identified as key practitioners who were likely to bolus patients if urine output was low. This was identified as a teaching and learning opportunity, and targeted resident education was established. In addition, site-based strategies to ensure daily weights were measured and charted, and reducing rate of standard infusions and locking off IVs were adopted as standard practices. Adoption of modern fasting guidelines and carbohydrate loading prior to surgery represented a significant change in practice for patients, health care workers (i.e., admission clerks), and health care providers (nurses, surgeons, anesthesiologists). Concerns from physicians were based on fear of having to postpone surgery for a patient who has eaten. Specifically, prior to ERAS implementation, these guidelines had not been adopted as standard of care. Strategies to address these barriers focused on creating clear education tools and a publicity campaign for patients that were applied across sites. Uptake of modern fasting guidelines with development of site-level policies in support of this was effective. Carbohydrate loading is undertaken to enhance insulin sensitivity and provides patients energy, from carbohydrates, and fluid. Patients mentioned that they enjoyed these guidelines. The challenges identified to carbohydrate loading related to timing, product selection, and the use of carbohydrate loading in diabetics given the lack of evidence on safety. To address these issues, data from EIAS was shared with leading anesthesiologists regarding safety and absence of impact on OR scheduling. These leaders supported the culture shift at their sites. Pain and nausea were flagged by patients and health care practitioners as clinical issues of importance. Patient feedback related to pain control was poor. Sub-themes in relation to pain control identified that there was no consistent approach to ordering oral, non-narcotic analgesics and optimal use and timing for patient-controlled analgesia (PCA). Strategies that were developed built upon pharmacy capacity to create standards of practice for oral analgesia inclusive of a transition strategy. Consistently asking patients about their pain management also became a standard practice. Nausea was a challenging symptom for patients and had an impact on eating and drinking after surgery. The adoption of around-the-clock Zofran, a nausea score, and Zofran availability in ward stock helped address issues with nausea.

Supporting patients was identified in a number of barriers and enablers to care. It was observed that patients play a major role in driving their clinical care related to nutrition, mobilization, pain and symptom control, and hydration. Patients wanted to be involved and engaged in their care from the time of diagnosis until after recovery. They wanted to know about ERAS and why compliance with the guidelines was important to support their own surgical journey. Although they were motivated to be discharged earlier, patients were concerned about what happens after discharge. Patients did not feel able to advocate for themselves, but expressed an interest in learning how to advocate for themselves through effective decision-making. They identified the role of stress throughout their journey. The use of perioperative counsel and support, as well as activities such as yoga, meditation, mindfulness, and exercise, were flagged as potential strategies to deal with stress. Almost 50% of the patients had colorectal surgery for cancer—in these patients, delays in test results and supports for patients with earlier stage cancers were identified as barriers. Timely follow-up with the surgeon and postoperative contact with an ERAS coordinator was felt to be valuable.

Education strategies for patients to enhance the uptake and impact of ERAS were identified repeatedly. Challenges related to outdated, conflicting, and confusing information across multiple sites. Prior to ERAS implementation, there was no education available on optimizing preoperative health. The mode of education (Web-based, books, videos, face-to-face meetings) was potentially a challenge. Strategies were adopted at the site and province levels to work towards timely and standardized preoperative teaching tools. Patients and families were involved in the education planning. Options to support rural patients and address issues related to language, cognition, and elderly patients have been identified, and work has begun at a provincial level. Patients wanted simplified materials, such as more pictures and chronologic timelines related to recovery. This development work was supported by a design student. A focus on the education materials was to ensure that patients understood expectations after surgery (i.e., eating, mobilizing, colostomy bag). Patients also valued education about when to ask for antiemetics, nutrition supplements, exercise, wound care, bowel continence, and the consequences of not following the guidelines. Further patient-centric research is planned to explore these issues.

Health care providers influenced the capacity in the system through their involvement in teams, their development of tools, and their role in communication and education. Health care providers identified barriers related to the culture of the environment in which they worked, including challenges related to changing long-held practices, and variable staff acceptance and uptake of the guideline by physicians and nurses. Resistance was identified in many forms, including late adopters, desire to see ERAS fail, and dwindling excitement following early implementation. Strategies to facilitate a culture shift included using data to gain staff buy-in, focusing on the evidence of patient benefit, allowing sites to customize the interventions, and having open discussions around expectations and challenges. The creation of site-based teams with identified and funded nurse coordinator positions, a physician champion, and working with a model of front-line ownership supported a culture shift. The provincial ERAS team had challenges initially in the absence of a dedicated project manager to align provincial activities across the six sites. In addition, early challenges included inconsistent project decision-making. Ultimately, support and alignment of decision-making at senior AHS levels addressed gaps and created an environment of support to enhance the capacity of front-line staff. Tools including order sets and protocols allowing standardization of practice reflected strategies that could be developed provincially and tailored to the local context. Audit of practice through EIAS not only was valued but also was flagged as burdensome. Issues relating to data included those of both data capture and management. Having a consistent strategy for communication within and across teams and for a variety of audiences was identified as an enabler to ERAS care.

AHS is a provincial organization with operational organization occurring at the hospital (site) level. Organizational capacity for ERAS was enabled by champions at the organizational level in the provincial ERAS team. Allocation of funding for ERAS nurse coordinators was vital for site-based involvement. In addition, organizational support for funding support for the ERAS® care system, including EIP and EIAS, allowed and supported the implementation. ERAS teams at the site and provincial levels worked in an ongoing fashion to develop, implement, and share strategies to remove barriers and enhance enablers to guideline adherence and best practice. These interventions were not explicitly measured or articulated. Rather, the practice changes needed to enhance compliance, to remove or reduce barriers, and to enhance facilitators were integrated in an ongoing fashion to evolving practice at each site, relative and responsive to local context.

The use of EIAS data by health professionals is a novel practice in the AHS health system. There were challenges with the volume of data, lack of electronic data collection processes, unreliable data collection tools, such as patient logbooks, and missing data. In addition, the nurse coordinators collected most the data, which was identified as not the best use of their skill set. Coordinated strategies to harmonize the electronic data capture with EIAS and help with data entry by health information clerks were useful in addressing this issue. A corporate approach to health professional education through the development of an annual ERAS symposium with invited international experts to share learnings and expertise locally was developed to address the education needs of health professionals involved in ERAS and to create capacity at the system level for ERAS spread, scale, and sustainment.

Across sites, there was no discernable pattern in the proportion of barriers and enablers identified or the nature of barriers and enablers related to sustainability considerations, such as culture, capacity, supportive environment, or clinical features.

**Fig. 6:**
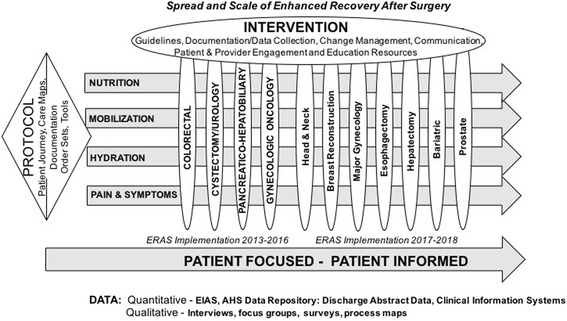
Model for spread, scale, and sustainment of ERAS: supporting system transformation

Building upon this data, we have created a model for spread, scale, and sustainment of ERAS in AHS (Fig. 6). The model builds upon evidence-based guideline implementation with support and resourcing for data collection and documentation, change management, communication, engagement, and education. “Mass customization” is facilitated through the development of protocols related to prioritized clinical care areas and includes the development of care maps, order sets, documentation strategies, and tools. The work is patient-focused and based upon ongoing qualitative and quantitative data capture. Strategies developed and identified with colorectal implementation can be applied across other surgical areas with streamlining of processes. Application of this model will support system transformation and allow broad-spread application.

Ultimately, the strategies adopted to address barriers and facilitators are reflected in change in compliance post implementation. Health provider practice did change over the 32 months of implementation, and practice change was sustained for the duration of the observation period based on ongoing measures of compliance with the ERAS® evidence-based guideline (Fig. 1). The greatest changes in compliance occurred in the preoperative and postoperative time periods where average compliance pre and post was 61.7 ± 12.8 to 84.5 ± 16.7% (*p* < 0.001) and 27.3 ± 10.3 to 53.0 ± 17.7% (*p* < 0.001), respectively. Compliance changes were similar across sites.

The implementation had a significant impact upon clinical and system-level outcomes, including LOS, complications, readmissions, and cost. The median LOS was 7 days (1–92 days) for pre-ERAS compared to 5 days (0–132 days) in post-ERAS (*p* = 0.009). The adjusted risk ratio was 1.71 (95% CI 1.09–2.68) for 30-day readmission, comparing pre-ERAS (22/126, 17.5%) to post-ERAS (65/677, 9.6%) patients [9]. The proportion of patients who developed at least one complication was significantly reduced, from (74/130, 56.9%) pre-ERAS to (315/695, 45.3%) post-ERAS, a difference in proportions of 11.7% (95% CI 2.5–21.0, *p* = 0.014) [9]. Severe complications, such as respiratory, cardiovascular, and renal and gastrointestinal complications, decreased by 9.2% (95% CI 2.86 to 15.47, *p* = 0.0002), 4.6% (95% CI −0.41 to 9.58%, *p* = 0.022), and 9.5% (95% CI 0.72 to 18.40, *p* = 0.025), respectively [9]. Surgical, infectious, epidural-related, and psychiatric complications decreased by 8.9% (95% CI 0.91 to 16.86, *p* = 0.015), 8.8% (95% CI 1.91 to 15.69, *p* = 0.0024), 0.4% (95% CI −1.87 to 2.65, *p* = 0.71), and 1.3% (95% CI −6.36 to 8.98, *p* = 0.73), respectively [9]. These impacts on clinical and health system outcomes were shown and aligned in both the EIAS data.

## Discussion

In this work, we report upon end-to-end implementation of an ERAS program, building upon the QUERI framework for implementation and using an established program for ERAS implementation across multiple sites within a single health system. We apply the TDF to help understand behavior at the individual and organizational levels. The TDF provides a method for systematically exploring barriers, enablers, and strategies to affect practice change. By using an established program, phasing in implementation, and using an integrated approach to mobilizing adoption of evidence-based practice, we have been able to extend and adapt process from early adopter sites to include the majority of centers performing colorectal surgery in AHS. We have applied the EIP and EIAS to support and enhance health care provider practice change and have demonstrated the value of real-time feedback of audit data to ERAS teams to inform and motivate practice change.

Although there are several reports of the impact of the ERAS care system on outcome [2, 23, 24], they typically report upon single-site, single-protocol (e.g., colorectal surgery) implementation. To date, end-to-end implementation in ERAS has not been described. In other jurisdictions, considerations of implementation of ERAS based on iKT, including an assessment of barriers and enablers, have been reported [25–27]. Mcleod et al. used a tailored approach to develop and implement an Enhanced Recovery evidence-based guideline rather than adopting an existing one because of potential limitations inherent in the guideline [25]. This process was undertaken at multiple sites, and an initial assessment of compliance was undertaken. In this study, a retrospective chart audit was undertaken to assess compliance with 18 identified ERAS interventions based on the evidence-based guideline that was locally developed [25]. Assessment of compliance was not repeated after implementation. Barriers and enablers were evaluated in this study, and that information was used to tailor interventions. Key features identified for success included identification of champions, strategies to facilitate communication and to share best practices, strategic management at an organizational level [26], use of standard order sets, and the role of audit feedback. These observations are in line with those seen in our implementation. This iKT work was not linked to an ongoing audit process or to specific outcome measures; however, they did document that using a tailored iKT strategy was helpful in adoption at multiple sites with the potential for use in other areas. In other work, enablers identified across the different medical professions included feasibility and alignment with current practice, standardization of care, good teamwork, and communication. Barriers included difficulty in adapting to change, lack of coordination between different departments, special needs of unique populations, limited resources, and rotating residents [27].

Through our ERAS implementation, we have identified and capacitated champions across multiple domains. Individuals at each organizational level have unique and critical roles to play in implementing and sustaining quality improvement [28]. The importance of champion coherence, external and internal relationship building, and the strategic management of AHS organizational-level visibility have been recognized locally as vital to the uptake and sustainability of ERAS [26]. Our recognition of this led us to the identification of the need for a vertically integrated model that links iKT at the patient, provider, and system levels, with each level acknowledging the need for integration and tailoring of interventions to address barriers and enablers to quality care. Our results provide support for the Behaviour Change Wheel model [29] by emphasizing the importance of focusing on capacity building for providers with training, communication, and motivation and creating a supportive environment with guidelines and fiscal measures, which ensure providers know what they need to do clinically and have the knowledge and skills to do this.

Early learnings identified that a focus on implementation and practice change was needed in the preoperative and postoperative time periods. It is during these stages of the patients’ surgical journey that there are more providers involved in the care of the patients. The identification of clinical themes including nutrition, mobilization, fluid management, and pain and symptom control allows the development of tools and processes (such as protocols, pathways, and order sets) to address barriers identified by health care providers that can be used more broadly across the system. The consistent finding of relevance of a standard approach to education and communication provides the impetus to link education for both patients and providers to an agenda that results in a quality strategy that may be generalized. Milne et al. have observed that quality criteria for acceptability, accessibility, appropriateness, equity, clinical effectiveness, and cost-effectiveness can only be truly addressed by a learning organization approach [30]. Organizational strategies that they have promoted include development of multidisciplinary training and learning environments, support of workplace learning, and modeling evidence- and knowledge-based practice [31]. Although this work has been undertaken in primary care, it has direct relevance to programs such as ERAS.

The use of data to drive practice change represents both a barrier and an enabler. In particular, Canada lacks a clinical culture that seeks and uses clinical performance data to drive improvement [32]. One of the largest practice changes that have been initiated with the implementation of ERAS across the six sites is the expected use of real-time data to guide practice change, and this is reflected in the barrier and enabler analysis. Brehaut et al. [33] describe 15 suggestions for optimizing effectiveness of practice feedback, which include those related to the nature of the desired action, the data available for feedback, the use of appropriate feedback display, and ability to deliver an intervention related to the feedback. The EIAS represents the “gold standard” for audit and feedback in colorectal surgery—it builds upon an evidence-based guideline, is informed by all cases, is timely, links visual displays and summary messages, is credible, provides feedback in a variety of ways, and allows the ERAS team to address barriers to care. However, we have also learned that there needs to be adequate support for data capture, including use of electronic data, data capture is duplicated, and data captured are not all used to inform practice change. In addition, the number and range of ERAS programs, with an aligned EIAS, available is currently limited at this time to colorectal, radical cystectomy, pancreaticoduodenectomy, and gynecologic/oncology [11, 34–36], but more areas of surgery are under development. There is also a cost associated with the ERAS programs. We have learned that development of a “minimal data set” and a revised audit process building upon evidence-based guidelines for best practice may be a way forward to address these gaps.

The ability to assess our qualitative data related to context is helpful as we consider strategies to spread and scale the ERAS innovation. While these data inform us of provider practices that warrant focus, they are less illuminating of what needs to happen at the patient and health system levels. Our approach to date has not targeted information gathering to address this. The alignment of practice change as it relates to patient support, involvement, outcome, and experience warrants further study. The alignment of practice change with outcomes that are relevant at the health system level, such as LOS, cost, complications, and readmissions, provides compelling evidence for decision-makers in terms of organizational prioritization and funding allocation. It is recognized that influences on sustainability include organizational context, capacity, processes, and factors related to the implementation of ERAS [37] and that these also warrant further evaluation. The application of a structured approach to this evaluation at the system level is required. Given the usefulness of applying the TDF in the current work, it provides a framework upon which it may be advanced in the realm of patient and system contexts.

Limitations of this study included a potential data bias from SharePoint as mostly the administrators and site coordinators posted documents. Only data from the first 12 months post implementation portion of the data coding were reviewed by a second reviewer. However, if the primary coder was unsure of what to code a quote, it was presented to the ERAS Knowledge Translation group. The provider survey did not have a response rate. Finally, quotes were difficult to stratify by site as our methods were not structured to measure across sites and because of the collaborative methods used by sites for knowledge mobilization.

## Conclusions

The application of the QUERI model and the TDF to system-wide implementation of an Enhanced Recovery After Surgery program for patients undergoing colorectal surgery has allowed us to detail processes and strategies to successful implementation across multiple sites. This systematic approach, linking research and practice, will inform the development of a model for spread, scale, and sustainability of Enhanced Recovery strategies more broadly across the health system. More work is required to detail and vertically integrate patient and system perspectives with the work that has focused on the health care provider based on an evidence-based guideline.

## Appendix 1

### Data analysis detailed process

1. Extract documents from SharePoint and documents from sites, project coordinators, emails, etc. and import into NVivo 11.
2. Organize documents into folders by theme and develop folders for new themes.
3. Organize documents within each folder and, where appropriate, move documents into sub-folders.
4. To understand the prominent themes in each folder, we created separate word clouds based on word frequency.
5. We created queries based on the results from the word clouds.
  - Example: implementation, patient, surgery.
6. Create word search barriers.
  - Use similar word definition (ex: barriers = obstacle, problem, issue).
7. Create word search enablers.
  - Use similar word definition (ex: enablers = aid, help, easy).
8. Decide on code structure and definitions (ex: site is UAH, FMC; provider is a nurse, surgeon; time frame is pre-ERAS before the first ERAS patient, 1–3 months).
  - See the coding structure sheet.
  - KT group discussion about structure.
9. Code barriers and enablers as such and add classification codes (site, provider, time frame, etc.).
  - Separate Excel spreadsheet for classifications.
10. Begin coding priority documents (ex: survey docs as requested by Ellen).
11. Begin coding priority sites through key word search (PLC and GNH).
  - May need to code “PLC or GNH” since some may not describe site, but are from 2013.
12. Begin coding searches “enablers” and “barriers.”
13. Begin coding key word search “data.”
  - Use exact word search “data.”
  - KT discussion regarding data.
14. Begin coding next priority docs (highlighted docs (LC#2, LC#1, TTTs, interviews, etc. as requested by Ellen).
  - KT discussion about coding definitions.
  - Add communication, logbooks, videos, team.
15. Pull matrix for time frame, site, and barriers/enablers.
  - Able to identify gaps in coding.
  - Request docs to fill in implementation gaps.
  - KT discussion—did not happen.
16. Add ERAS 22 elements to the coding structure.
17. Begin the framework of Michie and Rubenstein in Word doc.
18. Ellen codes first-order and second-order coding from quotes in Word doc.
19. Clean up the document set of duplicates.
  - Lots of documents imported throughout the coding process, need to ensure no duplicate coding of documents.
  - All sources on ERAS laptop only.
20. Transfer Word doc of framework and classification coding to Excel spreadsheet to facilitate large data set.
21. Scrap Word document for first- and second-order coding—use Excel only
  - Remove Ellen’s theoretical coding from Excel and clean Excel.
22. Remove Michie and Rubenstein coding, remove site and time frame coding.
23. Add themes picked up by coder (Caroline) to Excel.
  - Education, culture, resources, tools.
  - Put remaining in the Other category.
24. Add clinical coding groups (diabetes, MFG, nutrition, etc.) to Excel.
25. Add Rubenstein coding again to Excel.
26. Move all colorectal after 18 months post-ERAS implementation out of multiguideline and into colorectal on NVivo 11.
  - Code LC#3, interviews, and meeting notes in Excel.
27. Add the QUERI framework (capacity building, supportive environment) to Excel.
28. Ensure Excel coding complete for all new coding structures, and remove old coding structures.
29. Barrier and enabler quotes were sub-themed in Word.

## Appendix 2

### Exemplar quotes from “Scale and Spread” themes (Table 4)

#### Nutrition

Barrier:

“GNH—biggest issue was feeding patients after soup/sandwich 1st day resulting to eat it all and then throw up.”

Enabler:

“We changed the medpass structure. The front line nurses told us how they wanted us to do it. Fewer doses, option of two cal vs ensure, options for patients. Patients cued nurses. Audited this manually and he looked in mars. 60% of patients getting full dose in day 1. Telling patients its like a medication, we had to change the format of how we were doing this, that’s the most change.”

#### Mobilization

Barrier:

“Mobilization—healthcare aids more involved in this, physio isn’t going to help, education will make a difference in charting. Nurses said that charting was difficult for partial hours. Nurses were given enablers, but not using. Nurses need to mobilize.”

Enabler:

“Every day after that I knew what was recommended up to four times a day, walk around my ward. I had a number of family and friends in to visit me…to heck with this let’s do some laps.”

#### Fluid management

Barrier:

“Continue to struggle with is our residents and fluid management postop, and I think that shows up in our compliance data that our residents are still chasing urine outputs and they’re still flooding patients with bolluses postop.”

Enabler:

“Recognize the large amount of fluid overload occurring in the colorectal population. The EIAS database allowed us to collect very detailed data relating to volumes of fluid given intra operatively and post operatively in the the patients recovery. daily weights showed us that our patients were gaining between 2-4 kilograms post operatively. This lead to discussions around strategies to reduce fluid pre-intra-post operatively to improve outcome. Strategies included; reducing bowel preparation or eliminating all together, decreasing pre-op fluid administration, reducing standard post operative fluid administration rates using a standardized SCM Order Set and reducing boluses in the postoperative period for low urinary output.”

#### Diabetes

Barrier:

“Most difficult are the kind of the pieces or the patients that don’t fit nicely into those international recommendations, so carb loading for diabetics, and all of those pieces where we actually have to do a little bit more research before we would just adopt this provincially as best practice or standard practice.”

Enabler:

“The diabetic protocol needs to be completely clear and the Dr’s should follow the same thing.”

#### MFG & CHO loading

Barrier:

“Territory of of dramatic change such as the Fasting Guidelines or the carb load, that’s where you start hitting multiple departments around the hospital and and the culture that follows behind them and that takes a high level or a high degree of change in communication.”

Enabler:

“Need to establish the kind of populations and cases ERAS and modern fasting guidelines were rolled out simultaneously and this caused a lot of trouble. Took a lot of work to educate and get people to realize fasting guidelines are different than ERAS—it would be a good idea to uncouple the ERAS protocols from modern fasting guidelines sitewide and province wide.”

#### Pain and symptom management

Barrier:

“I had to pain crisis when one of the nurses did not wake me up to give me my medication that I needed to keep my pain under control. This set me back a bit. Keeping pain under control is as important as eating, drinking, walking and chewing gum.”

Enabler:

“Recognize how large of issue PONV was in the Colorectal population. Using the EIAS database as an instrument for quality improvement pointed various site in various different directions. PONV rate were very high at our site. We decided to break down PONV into Pre-op/Intra-op/Post-op treatment. we looked at current practice and the literature to see what were possible strategies to improve outcomes in these areas. Pre-operatively we identified that we were not performing standardized risk assessment for PONV. The Apfel screening score was added to the Pre-op in clinic Order set for screening of patients. Aprepitant PO was added to the pre-op order set. Intra-op the EIAS database collects information on whether or not the patient received intra op anti emetic. Our rates of administration have increased through meeting with site Anesthesia. Currently a literature review is in process to help identify a nausea score that is tested for reliability and validity. Currently we are still using the ten point visual analogue scale which was a new strategy for capturing nausea rates and efficacy of medication administration for the treatment of Post Operative Nausea and Vomiting.”

#### Thromboprophylaxis

Barrier:

“Have issues at UAH where pt comes out and the default the next dose to 830 the next morning then will get a double dose within 12 hours and more complicated with epidural. Problem with standard med admin time. It’s a pharmacy issue at UAH site. Not just the program that gives drug, look at work load and its changes workload they did not want to sign on to 2030 dosing time. IT is a patient safety issue.”

Enabler:

“In the meantime pharmacy is trying to set up a caution to look at the time of last dose. RAH has no issue because they have VAX. Very beneficial for ERAS.”

#### Education (provider)

Barrier:

“We also had a little bit of a different experience. We have a nonlocalized colorectal surgery patients going, that just means that patients go to three different units so it did expand the scope of the staff that we had to train and orient. And then, also, there were some new skill and expectations, so, really, it is a a eductation piece. It's not vastly different than standard practice but it is…there has to be discussion and there has to be education and there has to be communication, coaching and support, so I think those things, having to pull that together do take time.”

Enabler:

“In terms of the surgical rounds and anesthesia rounds and even the teaching for nurses and others, we have a library now of those kinds of presentations for the next sites to build on and adapt.”

#### Education (patient)

Barrier:

“Feedback from patients that language, format, presentation not consistent with messaging. This is a disadvantage to patients because it creates inconsistency.”

Enabler:

“My surgery pal app is a tool to point patients to the information they need in specific phases of care and to send notifications to patients as reminders. (8 notifications over 2 weeks). Videos are a tool, enhanced recovery after surgery on myhealthalberta, log and classroom teaching vs. one-to-one teaching.”

#### Team (site)

Barrier:

“Surgeons not buying into the program gets in the way—telling people things in the office vs. in hospital it ends up being conflicting advice. Now nurses ask “what has your doctor told you” as the starting point. Consistency is a challenge because the nurses and docs aren’t on the same page—it causes mistrust in the patient. Patient needs to know they are at the heart of it and that the team is working together around them to make their outcome a good one.”

Enabler:

“Create a multidisciplinary site team with various perspectives linked into the provincial team o Use a core site team to help break interdisciplinary silos and work together; meet biweekly to achieve an active, continuous process (e.g., review reports, prioritize what to work on, discuss issues, confirm targets & goals for improvement); meet early in the day with surgeons and anesthetists to not take away from their operating room time.”

#### Team (provincial)

Barrier:

“There were missed opportunities for the ERAS Project provincial project team (e.g., attending site meetings) to learn more about local collaborative practice changes and quality improvements within integrated multidisciplinary teams to inform other projects.”

Enabler:

“The Grey Nuns ERAS Steering Committee will continue to work with the provincial team to guide the implementation of both the tracking system and system level changes to implement ERAS across the Surgical Program. Weekly conference calls are planned with the provincial team project manager and an ERAS coach.”

#### Resources

Barrier:

“The discussions didn’t go super because we were in the midst of dealing with CoACT implementation, work force transformation, I don’t know, you know, bed rejigging, you know, in zones. People were under monetary pressures, you know, bugetary pressures, so there was a lot of things happening, and so many sites just said “no, not me”, you know, “we’ll do, you know, we’ll go the second round; yeah we’re intersted but not now.”

Enabler:

“Increase project planning time up front and secure adequate staff resources consistent with Institute of Health Improvement recommendations in order to balance flexible project management methodology with leadership and change management including a) dedicated day to day project coordination, b) clinical leadership, c) project management, d) administrative/leader sponsorship, e) data/technical analysis.”

#### Tools

Barrier:

“Logbooks: there are two different purposes that may be conflicted or at least not well-aligned: 1) source to collect data to support database and understand compliance and 2) patient tool. For the former, it is mixed purpose. Patient is sometimes not sure why they should fill it out.”

Enabler:

“Patients like taking materials home to process and see/learn—you process only so much at the start and it is good to have materials that accompany you home for when you aren’t stressed. A cheat sheet is helpful because people have something to go back to that is a reminder for what needs to be done.”

#### Data/EIAS

Barrier:

“Frustration with data, need to do some back work and it is not sustainable. There is going to be a huge volume of patients. Can’t fully rely on data because we’re in a transition but we will persist through it.”

Enabler:

“Develop a query function for SCM that gathers available data elements for data abstraction. Additionally, updating charting procedures to include daily nausea score and length of incision would make 95% of ERAS data elements available on SCM, with the remaining 5% continuing to come from the ERAS logbook. A query function would reduce the time needed for chart review by PC or clerk (if assigned).”

#### Culture

Barrier:

“There’s a lot of things that had to happen, a lot of culture that had to change or or at least make that changes that the culture could change along with them and the thing that I found the most the most difficult was the perception people had about, well we’ve been down this road before and it’s never worked before, and a prime example of that was the Modern Fasting Guidelines, and a number of people I ran into the hallways said “Wow, you know, patient’s, when you tell them they can have apple juice, well, the next thing you know they’re going to be having a Big Mac for breakfast or whatever” and so everybody was kind of set up looking for this to backfire and to a certain extent we have had our challenges with Modern Fasting Guidelines.”

Enabler:

“At each site—a lead surgeon, lead anesthetist in a ‘triad’ with a site coordinator to tackle barriers from different directions, talk to peers, manage relationships. Champions need to be progressive, open minded to changes in practice, not stuck in their ways.”

#### Communication

Barrier:

“Message/communication should come from the site leadership or Surgical/Anesthesia Chief. Although posters were posted across the site, memo sent to the physicians’ offices and discussions have been held about implementation of fasting guidelines across the site, on day of implementation many anesthetist/surgeons still were not aware of the memo or communication and hence many opted out.”

Enabler:

“Provide guidance to site teams, project teams and project managers on the multiple layers and levels of complex project communication and establish robust communication plans and practices at the beginning of a project that can be sustained.”

#### Supporting patients

Barrier:

“I think there is really nothing stressed about the mental, psychological aspects of it. For colorectal patients, you are going through a tumultuous time in your life… Not just colorectal but I think any cancer patient, you are going wow!… faced with your own mortality…”

Enabler:

“I felt like I was partner. It felt like there was a lot of me. It was good. 276) I felt like I was the one really healing myself, because of all the walking, food intake, how much I wanted to eat. I felt like I was an integral part in it. 277) They were helping me, encouraging me, providing me… It was a partnership.”

#### Other

Barrier:

“Conformity across sites just for the sake of conformity can hinder/harm patients. Sites are different; conform but tweak.”

Enabler:

“We actually—so the big thing about ERAS is it really isn’t a menu. So that is the way we’ve always been taught it. It is not a menu, you can’t pick and choose what you want to implement. Uh definitely, there is various things you are focusing on like right now we have our 5 scorecard items. But you really have to implement all 22 at once. Because you don’t really know how they interact with each other. So prime example, uh if I decide that I am going to let’s say IV fluids. I am going to give my patient a bowel prep and then I am going to flood them when they come in. Because I want to try to catch up on that bowel prep. Uh and then that, so IV fluid goes right from when they start the IV right to their discharge. And it can impact so many different things. So if I fluid overload my patient because I gave them a bowel prep; and I think they are dry. It impacts everything else. It can impact their complications. It can impact obviously their length of stay. It can impact uh morbidity mortality… ambulation… Do you see how one element can impact all other elements? So they are so tightly together that you can’t pick and choose one element and say… you know.”

## Abbreviations

AHS: Alberta Health Services
EIAS: ERAS Interactive Audit System
EIP: ERAS Implementation Program
ERAS: Enhanced Recovery After Surgery
iKT: Integrated knowledge translation
KT: Knowledge translation
LOS: Length of stay
MFG & CHO: Modern Fasting Guideline and Carbohydrate Loading
PDSA: Plan-Do-Study-Act
QUERI: Quality Enhancement Research Initiative
SCN: Strategic Clinical Network
TDF: Theoretical Domains Framework
TTT: “Train the Trainer”
